# What have worm models told us about the mechanisms of neuronal dysfunction in human neurodegenerative diseases?

**DOI:** 10.1186/1750-1326-4-38

**Published:** 2009-09-28

**Authors:** Dawn Teschendorf, Christopher D Link

**Affiliations:** 1 Institute for Behavioral Genetics, University of Colorado, Boulder, CO, 80309, USA

## Abstract

The nematode worm *Caenorhabditis elegans *has become an intensely studied model organism, and worm studies have made significant contributions to developmental biology and other fields. The experimental advantages of *C. elegans*, particularly its simple anatomy, optical transparency, short lifespan, and facile genetics, have also led researchers to use this model to investigate neuronal cell degeneration and death. Worm studies of neurodegeneration can be divided into two general classes: studies in which mutations of *C. elegans *genes lead to neuronal dysfunction and death, and studies in which external manipulations (e.g., chemical treatments or introduction of engineered transgenes) are used to induce neurodegeneration. For both types of studies the primary approach has been to use forward genetic, reverse genetic, or candidate gene approaches to identify genes that modify neurodegeneration. The ease and relatively low cost of *C. elegans *propagation also suggests a role for these *C. elegans *models for compound screening. An excellent review has been previously published that summarizes much of the work done on mutationally-induced neuronal death in *C. elegans *[1]. This review focuses on studies that have attempted to model specific human neurodegenerative diseases using transgenic approaches. These studies have given us a variety of insights into the specific disruptions of cellular processes that may underlie human neurodegenerative diseases.

## Are worm models of human neurodegenerative diseases a good idea?

Although invertebrate model systems (e.g., *C. elegans *or *Drosophila*) have significant experimental advantages, it is not self-evident that they are good approaches to study human neurodegeneration. As detailed below for *C. elegans*, there is strong support for the conservation of basic neuronal cellular functions between invertebrates and vertebrates. The critical question is therefore whether the specific neuronal cellular functions that are directly perturbed in neurodegenerative diseases are also conserved between these model systems and people. Given that the causal molecular/cellular insults underlying neurodegenerative conditions are not known (or at least controversial), the answer to this question is unclear. It is clear, though, that expression of specific human proteins linked to neurodegeneration (e.g., β-amyloid peptide, tau, α-synuclein, etc.) leads to cellular toxicity in worms and flies. Minimally, invertebrate models should give us insight into (at least some) toxic activities of disease-relevant human proteins. The relevance of neurotoxic mechanisms established in worm or fly models necessarily need to be validated in mammalian systems. Thus, it is perhaps best to view worm models of neurodegeneration as "scout" systems that have the potential to generate important findings regarding mechanisms of neurotoxicity, with the caveat that all findings are probably provisional until extended to mammalian systems.

A major challenge in generating a worm neurodegeneration model is obtaining evidence that any observed pathology is specific and disease-relevant. One such piece of evidence for transgenic models is the demonstration that disease proteins containing mutations associated with familial forms of the disease are more toxic than wild type when expressed in worms [e.g., α-synuclein containing familial Parkinson's disease (PD) substitutions should be more toxic than wild type α-synuclein]. In addition, demonstrating that a disease model shows the same spectrum of neurotoxicty (e.g., preferential toxicity to dopaminergic neurons in PD models) and/or similar cell pathology (e.g., formation of Lewy body-like inclusions in PD models) as observed in the human disease also strengthens the case for the relevance of a model. It is reasonable to assume that worm models with cellular (or molecular) pathology more similar to the human disease have a stronger "presumption of relevance".

The strongest argument for developing worm models of neurodegeneration is that worms are amenable to unbiased genetic approaches, classically based on identifying forward mutations that modify the phenotype presented by a given model. In recent years this approach has been extended by using reverse genetic techniques such as RNA interference (RNAi). The value of these unbiased approaches lies in the two possible outcomes of a successful screen: either a gene previously linked to the disease is found, or a novel interactor is identified. In the first case the result strengthens previous findings, in the later case new insights may be gained. (Possibility #2 is a corollary of a creed in the field: genetics is a way to answer questions you weren't smart enough to ask in the first place.) Of course, novel findings from worm models require extra skepticism and validation.

Additional advantages of worm models include the short lifecycle/lifespan and the ability to generate transgenic strains relatively rapidly. At 20°C, the most common culture condition for *C. elegans*, the life cycle takes approximately 4 days and the mean lifespan is about 17 days. Thus, strains containing heritable (although typically unstable) transgenes can be constructed by microinjection in two weeks. (Derivation of completely stable, chromosomally integrated transgenes typically takes another month.) Combined with the ability to readily freeze and recover transgenic strains, it is feasible to generate large collections of strains expressing related transgenes [e.g., a series of engineered mutations in a protein or promoter region, a collection of Green Fluorescent Protein (GFP) reporters for a set of related genes, etc.]. This capacity allows comparative studies that would be very expensive and time-consuming to do in mice.

Although typically propagated on small agar-filled Petri plates, *C. elegans *can also be grown in microtiter wells, suggesting that worm models could be particularly appropriate for high-throughput drug screens.

## Relevant neuroanatomy of *C. elegans*

Adult *C. elegans *hermaphrodites contain only 302 neurons, and the complete pattern of synaptic connections has been reconstructed by serial electron microscopy. Neuronal classes include chemosensory, mechanosensory, and thermosensory types; 75 motor neurons innervate the body wall muscles (excluding the head); 56 of these are cholinergic and 19 are GABAnergic. *C. elegans *larvae contain 4 serotonergic and 8 dopaminergic neurons. Formation, trafficking, and release of synaptic vesicles in *C. elegans *is highly conserved, employing many of the same proteins as are used in mammalian neurons. Because *C. elegans *is transparent throughout its life cycle, GFP fusions have been extensively used to visualize specific neurons and synapses in living animals. In some instances, neuronal death can be directly observed in living worms by the appearance of vacuolated neurons. More generally, GFP tagging of specific neurons allows observation of neuronal cell dystrophy or loss throughout the lifetime of the animal.

There are limitations to studying neurodegeneration in *C. elegans*. Worms do not have myelination or an active immune system, so they are presumably not appropriate for some neurodegenerative conditions such as multiple sclerosis. Practically, worm neurons are small and difficult to patch clamp, although recordings can be made from single identified neurons [2]. RNA interference (RNAi), a particularly useful tool in *C. elegans*, is often ineffective in neurons, necessitating the introduction of additional mutations to enhance neuronal RNAi efficacy.

## What can we expect to learn from worm models of neurodegeneration?

Essentially all of the major age-associated neurodegenerative diseases (e.g., Alzheimer's, Parkinson's, ALS, etc.) have been linked to accumulation of specific proteins in the CNS. The neurodegenerative process in these diseases can be viewed as having three phases: accumulation of the toxic protein, toxic insult to neurons, and neuronal dysfunction and death. In theory, components in any of these phases can be identified by the identification of modifier genes in worm models of neurodegenerative diseases. This review will focus on a subset of *C. elegans *studies to illustrate how worm neurodegeneration models have been used to identify components of these phases of neurodegeneration. An inclusive list of studies employing transgenic *C. elegans *strains to study specific neurodegenerative diseases is shown in Table 1.

**Table 1:** *C. elegans *transgenic models for human neurodegenerative diseases.

**Neurodegeneration-associated protein**	**Transgene expression**	**Promoter Sequence**	**References**
Huntingtin::polyQ	chemosensory neurons	*osm-10*	Faber et al [3]
	mechanosensory neurons	*mec-3*	Parker et al [11]
	muscle	*unc-54*	Wang et al [19]
			
DRPLAP::polyQ	muscle	*unc-54*	Yamanaka et al [18]
			
GFP::polyQ	muscle	*unc-54*	Saytal et al [14]
	pan-neuronal	*rgef-1*	Brignull et al [82]
			
α-synuclein	pan-neuronal	*aex-3*	Lakso et al [21]
	dopaminergic neurons	*dat-1*	
	dopaminergic neurons	*dat-1*	Cao et al [22]
	dopaminergic neurons	*dat-1*	Kuwahara et al [23]
	pan-neuronal	*unc-51*	Kuwahara et al [25]
			
α-synuclein::GFP	muscle	*unc-54*	Hamamichi et al [26]
α-synuclein::YFP	muscle	*unc-54*	van Ham et al [27]
			
β-amyloid peptide	muscle	*unc-54*	Link [31]
	inducibe muscle	*myo-3*	Link et al [38]
	inducible pan-neuronal	*snb-1*	Wu et al [39]
			
tau	pan-neuronal	*aex-3*	Kraemer et al [50]
	mechanosensory neurons	*mec-7*	Miyasaka et al [57]
	pan-neuronal	*rgef-1*	Brandt et al [58]
			
SOD1	muscle	*myo-3*	Oeda et al [60]
	heat shock inducible	*hsp-16.2*	
	pan-neuronal	*snb-1*	Wang et al [61]
			
SOD1::YFP	muscle	*unc-54*	Gidalevitz et al [62]
			
LRRK2	pan-neuronal	*snb-1*	Saha et al [63]
			
mouse prion protein	muscle	*unc-54*	Park and Li [64]

### Polyglutamine repeat diseases

In 1999, Hart and colleagues described a *C. elegans *Huntington's disease (HD) model based on expression of a huntingtin fragment (exon 1) containing a 150 repeat polyglutamine (Ht-Q150) expansion [3]. Expression of this huntingtin fragment was driven by the *osm-10 *promoter, resulting in expression specifically in (non-essential) sensory neurons. Ht-Q150 expression was found to induce both neuronal dysfunction (demonstrated by the inability of chemosensory neurons to take up fluorescent dyes) and eventual death of chemosensory neurons (under conditions of *osm-10*::GFP co-expression). The apparent toxicity of Ht-Q150 was age-dependent but relatively mild, such that 13% of transgenic worms had dye uptake abnormalities at day 8 of development (mid-adulthood). This transgenic phenotype was subsequently used to identify mutations that enhanced Ht-Q150 toxicity, leading to the identification of *pqe-1 *[4]. Loss-of-function mutations in *pqe-1 *were found to dramatically enhance Ht-Q150-dependent chemosensory neuron death. *pqe-1 *encodes a nuclear protein rich in glutamines and prolines that also contains a conserved exonuclease domain (although PQE-1-homologous protein exist in mammals and flies, these proteins do not appear to contain glutamine/proline-rich domains). Given the cellular distributions of PQE-1 and Ht-Q150, these authors argued that wild type PQE-1 protein may protect from Ht-Q150 toxicity by competing for proteins sequestered by Ht-Q150.

The *osm-10*/Ht-Q150 model has also been used to investigate the roles of specific histone deacetylases in regulating huntingtin polyglutamine (Ht-polyQ) toxicity [5]. Studies in multiple models have demonstrated that expanded polyglutamine huntingtin can sequester CREB-binding protein (CBP) and its associated histone acetyltransferase activity [6,7]. Inhibition of histone deacetylase activity can counter this effect and subsequently reduce Ht-polyQ toxicity [8,9]. The ability to knock down specific gene expression in *C. elegans *by a simple feeding RNA interference procedure [10] allowed Bates et al to quickly assess the roles of 8 specific histone deacetylases in Ht-polyQ. Interestingly, knock down of one deacetylase (*hda-3*) reduced polyQ toxicity, while knockdown of the others increased toxicity as expected. Introduction of loss of function mutations for *hda-1*, *hda-4*, and *sir-2.1 *similarly enhanced Ht-polyQ toxicity.

A conceptually similar model for HD was developed by Parker et al [11] using the *mec-3 *promoter to express an N-terminal 57 residue fragment of huntingtin (with or without expanded polyglutamine repeats) fused to Green Fluorescent Protein (Ht-polyQ::GFP). The *mec-3 *promoter is active in 10 (non-essential) neurons, including the six touch receptor cells. Ht-polyQ::GFP toxicity was assayed by measuring touch responsivity in individual transgenic worms. As observed in the *osm-10 *model, increasing the number of polyglutamine repeats in the transgene led to increased deficits in touch sensitivity. Transgene-induced touch insensitivity was not associated with death of the touch cells, but could be associated with visible morphological abnormalities in touch cell axons. This model was subsequently used to demonstrate that over-expression of the *sir-2.1 *deacetylase could protect against Ht-polyQ::GFP toxicity [12]. This result was extended by demonstrating that resveratrol, a demonstrated activator of sirtuin acetylases, could protect against Ht-polyQ toxicity in both worm and neuronal culture models. Interestingly, *sir-2.1 *or resveratrol protection in the *C. elegans mec-3*/Ht-polyQ model was dependent on the FOXO transcription factor *daf-16*, suggesting that this protection was not due to reversal of Ht-polyQ effects on histone acetylation *per se*. The *mec-3*/Ht-polyQ:: model has also been used to demonstrate a protective role for *hipr-1*, a homolog of the HIP1 huntingtin-interacting protein, against Ht-polyQ toxicity [13].

Perhaps a more general approach towards understanding polyglutamine repeat toxicity was initiated by the Morimoto lab, which examined the effects of short (Q19) and long (Q82) polyglutamine repeat lengths fused directly to GFP and expressed in *C. elegans *body wall muscle cells [14]. Expression of GFP::Q82 resulted in aggregate formation and induction of heat shock proteins. Subsequent studies with a series of YFP-polyQ fusions demonstrated a narrow threshold of polyQ repeat size (35-40) for induction of aggregation and toxicity [15]. Examination of transgenic worms expressing threshold-level glutamine repeats (Q40) demonstrated a strong age dependence for aggregation as well as significant individual variability. This model system was employed in an elegant series of studies to demonstrate that formation of polyQ aggregates generally disrupted protein homeostasis [16]. Introduction of an aggregation-prone YFP::polyQ transgene into a collection of temperature-sensitive missense mutations was found to dramatically enhance the phenotypes of these mutants, while conversely the presence of these missense mutations was found to enhance the aggregation of YFP::polyQ. These studies are consistent with a general "chaperone depletion" model that posits that aggregating protein toxicity results from competition for limited components of the protein homeostasis machinery. Indeed, a large scale RNAi screen for modifiers of YFP::polyQ aggregation identified a large set of genes involved in protein folding or degradation that increased polyQ aggregation when their expression was knocked down [17].

Additional *C. elegans *polyQ models have been generated with muscle-expressed polyglutamine repeats associated with either 17 amino acid residues derived from the dentatorubural pallidoluysian atrophy protein (DRPLAP) [18] or huntingtin exon 1 [19]. The former model has been used to demonstrate protective effects of *C. elegans *p97 homologs CDC-48.1 and CDC-48.2, while the latter model was used to show ubiquilin protection against polyQ toxicity. The muscle Ht-polyQ model has recently been used to implicate mitochondrial fission/fusion in huntingtin toxicity [20].

The studies described above illustrate the advantages (and disadvantages) of *C. elegans *neurodegeneration model systems, and the type of findings that can be made. The ability to undertake unbiased forward genetic screens enabled the identification of a novel gene involved in Ht-polyQ toxicity (*pqe-1*). However, vertebrate homologs of *pqe-1 *do not contain the relevant glutamine/proline-rich portion of this protein, so the direct relevance to HD is unclear. The optical transparency and short lifespan of *C. elegans *readily allowed the demonstration of an age-dependence for polyQ aggregation, something that is difficult to do directly in other model systems. Similarly, the existence of a large collection of characterized *C. elegans *mutations enabled the important demonstration of polyQ aggregation-dependent perturbation of protein homeostasis. The technically simple feeding RNAi protocol has enabled a number of candidate genes (e.g., *hda-1*, *hda-4*, *sir-2.1, hipr-1, cdc-48.1,2 and dpr-1*) to be implicated in *in vivo *polyQ toxicity. As is the case in other model systems, however, the use of different transgenic constructs by different research groups does confound making direct parallel between some studies. For example, it is unclear if toxicity in the neuronal expression *osm-10*/Ht-Q150 model is due to the disruption of protein homeostasis, or if the huntingtin sequences themselves play an important role.

### Models of α-synuclein toxicity

α-synuclein is a major component of Lewy bodies found in dystrophic dopaminergic neurons in Parkinson's disease (PD), and α-synuclein mutations have been found to be casual in a relatively small number of familial PD cases. *C. elegans *models of synucleinopathy have been established with human α-synuclein expression in either dopaminergic neurons [21-23] or pan-neuronally [21,23]. Lasko et al observed movement deficits in worms with pan-neuronal expression of wild type or A53T α-synuclein, while Kuwahara did not observe any phenotypic effects of pan-neuronal wild type, A30P, or A53T α-synuclein expression. (These differences may be a result of the specific promoters used in the transgene constructions; Lasko et al used the *aex-3 *promoter, while Kuwahara et al used the *unc-51 *promoter). However, all three groups have reported loss of either dopaminergic neuron cell bodies or dendrites when α-synuclein expression is driven by the dopaminergic-specific *dat-1 *promoter. Kuwahara et al also observed preferential neuronal dysfunction in worms expressing mutant α-synuclein (A53T or A30P) relative to wild type, measuring either dendritic loss or deficits in behaviors (slowing upon food sensation) known to be dopamine-dependent. The *dat-1*/wt α-synuclein model of Cao et al was used to demonstrate the protective effects of the chaperone protein torsin A [22] and Rab1, a protein believed to function in Golgi vesicular trafficking [24]. The pan-neuronal α-synuclein model of Kuwahara et al has been used in a large-scale feeding RNAi screen to identify enhancers of α-synuclein toxicity, leading to the identification of a number of genes (e.g., *apa-2*, *aps-2*, *eps-8 *and *rab-7*) involved in the endocytic pathway [25]. Studies of *C. elegans *α-synuclein models have thus strongly supported the link between α-synuclein toxicity and intracellular (synaptic?) vesicle trafficking.

*C. elegans *models have also been constructed to look for genes specifically influencing α-synuclein aggregation, following the approach initially developed for polyQ-induced aggregation [17]. The Caldwell and Nollen groups both demonstrated that α-synuclein::GFP (or YFP) fusion protein expressed in *C. elegans *muscle cells leads to the formation of visible fluorescent aggregates, as previously observed for GFP::polyQ ([26,27]. Hamamichi et al initially tested 868 "hypothesis based" RNAi clones for enhancement of α-syn::GFP aggregation. (This screen was sensitized by co-expression of TOR-2 to reduce initial aggregation levels.) Twenty genes were ultimately identified that increased α-syn::GFP aggregation when their expression was knocked down early in development by feeding RNAi. Seven of these genes were tested for effects on α-synuclein toxicity (not necessarily equivalent to aggregation) by transgenic overexpression in the *dat-1*/α-synuclein model; five were found to partially suppress dopaminergic neuron loss. Interestingly, these validated aggregation/toxicity enhancer genes included the vacuolar assembly/sorting protein *vps-41 *and the autophagy-related *atgr-7*. van Ham et al similarly screened for genes that increased α-syn::YFP aggregation after RNAi knockdown, in this case using the "whole genome" Ahringer feeding RNAi library, which contains ~15,000 clones (~85% of the genome represented). 80 genes were identified, with an over-representation of lipid- and vesicle- associated genes. For three of these genes (*sir-2.1*, *lagr-1*, and *ymel-1*) suppression of α-syn::YFP aggregation was independently confirmed using genetic loss-of-function mutations.

Perhaps the most interesting result from these two large-scale screens is that essentially no overlap was identified between genes that suppressed GFP::polyQ and α-syn::GFP aggregation. This observation is consistent with results from yeast studies and supports the view that aggregation of different disease-associated proteins is not equivalent. However, it should be pointed out that there is also no overlap in the genes identified in the Hamamichi et al and van Ham et al studies. This lack of overlap could be the result of technical differences between the two screens, and in fact non-congruence between similar *C. elegans *RNAi screens is not uncommon (e.g., [28,29]). Large scale RNAi screens in *C. elegans *appear to have an inherent variability, and thus negative results from these screens should be interpreted cautiously.

### Models of β amyloid peptide toxicity

The β-amyloid peptide (Aβ) is a primary component of senile plaques found in the brains of Alzheimer's disease (AD) patients, and the existence of mutations in the gene encoding Aβ (Amyloid Precursor Protein, APP) in a subset of familial AD cases argues for a causal role for this peptide in this disease. Aβ has also been found to accumulate in muscles of patients with Inclusion Body Myositis, a debilitating myopathy [30]. Initial attempts to use *C. elegans *to understand Aβ toxicity employed the *unc-54 *promoter to constitutively express a signal peptide::Aβ minigene in body wall muscle [31]. Transgenically-expressed Aβ was found to accumulate intracellularly in muscle cells, and result in an age-dependent paralysis phenotype. This intracellular Aβ was able to form amyloid dye-reactive deposits with a fibrillar ultrastructure (i.e., amyloid deposits) [32]. Single amino acid substitutions (e.g., Leu^17^Pro) were identified that blocked amyloid formation in this model but did not reduce toxicity, suggesting that amyloid itself is not the toxic species [33]. The HSP-16 family of small heat-shock proteins was found to be induced by and co-immunoprecipitate with Aβ in this model [34], and ectopic expression of HSP-16 could suppress Aβ toxicity [35]. Cohen et al [36] used the *unc-54*/signal peptide::Aβ 1-42 model to investigate the roles of two important modulators of *C. elegans *lifespan, the insulin growth factor 1-like signaling (ILLS) pathway and heat shock factor (HSF), in Aβ toxicity. RNAi knockdown of either *daf-16 *(the FOXO transcription factor that controls gene expression downstream of ILS) or *hsf-1 *enhanced Aβ toxicity, while activation of DAF-16 (via RNAi knockdown of *daf-2*, a negative regulator of *daf-16*) suppressed toxicity. Bacterial deprivation, a form of dietary restriction that extends lifespan in *C. elegans*, has also been found to reduce Aβ (and polyQ) toxicity in a HSF-dependent manner [37].

Overall, the results of studies with worms with constitutive muscle expression of either Aβ or YFP::polyQ are similar, and suggest that in both cases the observed toxicity primarily results from a general perturbation of protein homeostasis (proteostasis). Interestingly, neither DAF-16 activation nor bacterial deprivation was observed to reduce the overall accumulation of Aβ, despite being protective. These results suggest that upregulation of the chaperone/protein folding machinery is protective either because this blocks the formation of some specific toxic form of Aβ (supported by the results of Cohen et al, who found changes in the aggregation state of Aβ correlating with toxicity) and/or because this compensates for a general depletion of chaperone capacity by deposition of Aβ aggregates. It is an open question whether the *unc-54*/signal peptide::Aβ 1-42 model is directly relevant to Alzheimer's disease, as the levels of intraneuronal Aβ accumulation in the brain are unlikely to reach the intracellular Aβ levels generated in this model. However, results from this model may be generally instructive in that they suggest that the age-dependence of AD on other neurodegenerative conditions may stem from age-dependent loss of the ability to maintain cellular protein homeostasis. It should be noted that this model may be more directly relevant to inclusion Body Myositis, given the parallels of intramuscular Aβ accumulation.

Transgenic worms have also been engineered to inducibly express Aβ upon temperature upshift, either in muscle [38] or pan-neuronally [39]. This temperature inducibility was engineered by using transgene constructs with abnormally long 3' untranslated regions, resulting in transgenic transcripts that are subject to degradation by the mRNA surveillance system. Introduction of these transgenes into worms containing a temperature-sensitive mutation in a gene essential for mRNA surveillance (*smg-1*) resulted in strains that had a ~5-fold increase in transgenic transcripts when shifted from the permissive (16°C) to non-permissive (23°C) conditions [37]. An advantage of this system is that, at least for engineered muscle expression, transgenic strains have been constructed that have wild type movement at the permissive temperature but become rapidly (~24 hr) and uniformly paralyzed upon temperature upshift. The reproducible phenotype of the *myo-3*/signal peptide::Aβ 1-42/long 3' UTR model has allowed straightforward quantification of the effects of treatments on Aβ toxicity, and has been used to demonstrate the protective effects of specific gingkolides [39]. This inducible model has also been used to demonstrate a role for autophagy in countering Aβ toxicity [40]. Microarray studies with this model identified AIP-1, a conserved protein thought to be a positive regulator of proteasome function [41,42], as an Aβ-induced protein that protects against toxicity by reducing Aβ accumulation [43]. Interestingly, a human homolog of AIP-1, AIRAPL, also reduces toxicity when co-expressed with Aβ.

Transgenic worms with pan-neuronal expression (signal peptide::Aβ driven by the *snb-1 *promoter) were found to have intraneuronal accumulation of Aβ [44] but relatively mild phenotypes, including altered chemotaxis to benzaldehyde and hypersensitivity to exogenous serotonin [39]. It is unclear if the apparent differences in the severity of phenotypes resulting from muscle or neuronal expression reflect differences in the manner by which *C. elegans *muscle and neuronal cells respond to Aβ, or simply quantitative differences in the effective expression levels resulting from the use of different promoters. McColl et al [45] have recently demonstrated by mass spectrometry that in the *unc-54*/signal peptide::Aβ 1-42 constitutive model the species of Aβ that accumulates is actually Aβ 3-42, likely due to signal peptidase cleavage after Ala^2 ^of the Aβ sequence. As the inducible models utilize the same signal peptide::Aβ minigene, it is likely that all of these models produce the truncated Aβ 3-42. The 3-42 form of Aβ is readily found in senile plaques, where the N-terminal glutamate residue is often converted to pyroglutamate [46]. However, McColl and colleagues did not detect pyroglutamate-modified Aβ in the transgenic worm model.

### Tauopathy models

Alzheimer's disease, as well as Pick's disease and some forms of frontotemporal lobar dementia (FTLD), are associated with intraneuronal accumulation of neurofibrillary tangles (NFTs) composed of the microtubule binding protein tau. Mutations in tau have been demonstrated to underlie familial FTLD linked to chromosome 17 (FTLD-17) [47-49], causally linking tau to some forms of neurodegeneration. Kraemer et al established a *C. elegans *tauopathy model by expressing wild type or FTLD-17-mutant tau pan-neuronally using the *aex-3 *promoter [50]. This model system replicated key observations of the human disease: accumulation of insoluble, phosphorylated tau, evidence of age-dependent neuronal degeneration and loss, a clear organismal phenotype (uncoordinated movement), and greater toxicity in worms expressing FTLD-17-mutant tau (V^337^M and P^301^L) than in worms expressing wild type tau. The uncoordinated phenotype of the *aex-3*/tau worms has been used for both classic forward genetic and RNAi-based reverse genetic screens, resulting in interesting (and perhaps unexpected) findings. Genetic screens based on chemical mutagenesis and positional cloning have identified two genes, *sut-1 *[51] and *sut-2 *[52], whose loss of function suppresses the tau-induced uncoordinated phenotype. *sut-1 *encodes a nematode-specific protein that binds Sm proteins and SmY, a small nematode-specific RNA of unknown function [53]. However, Kraemer and Schellenberg were able to use yeast two-hybrid and genetic studies to demonstrate that *sut-1 *interacts with UNC-34, a member of the conserved Ena/VASP protein family. They speculated that this interaction may modulate actin dynamics and thereby ultimately suppress tau pathology. *sut-2 *is a zinc finger-containing protein homologous to yeast Nab2 and human ZC3H14. Nab2 has demonstrated roles in the nuclear export of mRNA [54], and both Nab2 and ZC3H14 have been shown to bind polyadenosine sequences [55]. Guthrie et al also found by two yeast hybrid studies that *C. elegans *protein ZYG-12, and its human homolog HOOK2, can interact with SUT-2. Given that HOOK2 is a component of aggresomes, these authors suggested that *sut-2 *suppression of tau pathology could involve either aggresome effects on tau accumulation or, conversely, tau effects on proper aggresome formation.

The *aex-3*/tau V^337^M model was used as the basis for a full genome feeding RNAi screen (16,757 RNAi clones assayed), looking for modifiers of the tau-induced uncoordinated phenotype [56]. Although no suppressor RNAi clones were found, 60 genes were eventually identified that specifically enhanced the tau-induced phenotype when their expression was knocked down by RNAi. These genes encoded proteins with a surprising range of functions, including phosphorylation, chaperone activity, neurotransmission and signaling, RNA processing, and various enzymatic functions. Seven of these genes (*sir-2.3*, *vap-1*, *lin-44*, *aex-1*, *acr-14*, *pxn-1*, and *mut-14*) were independently tested by introducing genetic loss-of-function mutations into the *aex-3*/tau V^337^M background; in all cases the enhancement of the uncoordinated phenotype was recapitulated. The range of identified enhancer genes may reflect a multitude of steps by which neurons normally counter either toxic protein accumulation (e.g., altering tau modification, aggregation, or degradation) or its downstream consequences. Alternatively, the apparent interaction of these genes with tau pathology could result from a rather indirect synergism in which a reduction of gene function that normally results in a phenotypically undetectable compromise of neuronal function (e.g., reduction of *acr-14*, one of the many acetylcholine receptors in *C. elegans*) nevertheless significantly exacerbates tau pathology in a perhaps additive fashion. In any case, the interacting genes identified by both forward and reverse genetic approaches with this model suggest potentially novel mechanisms of tau pathology that warrant investigation.

Tau pathology has also been engineered in *C. elegans *by expressing wild type and FTLD-17-mutant tau specifically in touch neurons, using the *mec-7 *promoter [57]. Transgenic expression of tau in the touch neurons resulted in an age-dependent loss of touch sensitivity, again with FTLD-17-mutant tau (P^301^L and R^406^W) showing enhanced toxicity. This model was used to test the effects of HSP70 overexpression (mild suppression of mutant tau toxicity), GSK-3 overexpression (mild enhancement of tau toxicity), or genetic blockage of apoptosis (no effect on tau toxicity). Brandt et al [58] examined the role of phosphorylation in tau toxicity by using the *rgef-1 *promoter to engineer pan-neuronal expression of wild type, pseudophosphorylated (10 specific kinase target serine residues changed to glutamate), or phosphorylation-resistant (10 specific kinase target serine residues changed to alanine) tau. Both wild type and glutamate-substituted tau were observed to have similar age-dependent uncoordinated phenotypes, although worms expressing the glutamate-substituted tau had higher levels of abnormal motorneurons. (The alanine-substituted tau also induced an uncoordinated phenotype, although this was hard to interpret because transgenic lines expressing this modified tau all had significantly higher levels of tau expression.) Neuronal death was not observed in either the *mec-7*/tau or *rgef-1*/tau models. However, in both models neuronal outgrowth abnormalities were observed. While this could be indicative of developmental effects of tau expression on axon pathfinding, similar abnormal outgrowth has been observed as a result of regeneration of broken axons [59], suggesting the alternative possibility that tau expression results in fragile axonal processes more susceptible to movement-induced breakage.

### Other transgenic models

Mutations in superoxide dismutase (SOD1) are the most common known cause of familial Amyotrophic Lateral Sclerosis (fALS). Oeda et al [60] generated the first *C. elegans *model of SOD1 toxicity by expressing wild type or fALS mutant SOD1 (A^4^V, G^37^R, or G^93^A), using either the muscle-specific *myo-3 *promoter or the heat-shock inducible *hsp-16.2 *promoter. While these transgenic worms were not reported to have discernable phenotypes under standard conditions, worms expressing the fALS mutant SOD1 were found to be preferentially sensitive to paraquat. Wang et al [61] engineered pan-neuronal SOD1 expression using the *snb-1 *(synaptobrevin) promoter, expressing both wild type and G^85^R fALS mutant SOD1. Expression of SOD1 G^85^R (fused to YFP or unfused), but not wild type SOD1, resulted in a clear age-dependent inhibition of locomotion. Transgenic worms expressing G^85^R SOD1::YFP also had visible fluorescent aggregates in neurons, allowing a full genome feeding RNAi screen in a sensitized *eri-1*; *lin-15b *genetic background. As observed in the RNAi screens of the *aex-3*/tau model, both expected (e.g., chaperone-related genes such as *hsf-1*) and unexpected (topoisomerase gene *top-1*, TGF β component *dbl-1*) were recovered as aggregation/toxicity enhancers. (One aggregation/toxicity suppressor was also identified but not described in this study.) Transgenic worms expressing SOD1::YFP fusions have also been constructed by Gidalevitz et al [62], who used the *unc-54 *muscle-specific promoter to express wild type and G^85^R, G^93^A, and truncated (^127^X) fALS mutant SOD1. As reported by Wang et al, fALS SOD1::YFP, but not wild type, formed visible aggregates. While transgenic worms expressing fALS mutant SOD1::YFP had relatively mild phenotypes, introduction of temperature-sensitive missense mutations (the same mutant alleles that are enhanced by YFP::polyQ aggregation) strongly exacerbated SOD1::YFP toxicity in an fALS mutant-specific manner. This result nicely complements the previous YFP::polyQ study by the Morimoto group, and supports the idea that misfolded proteins (i.e., destabilized missense mutant proteins and fALS mutant SOD1) compete for limiting protein homeostasis machinery.

Mutations in leucine-rich repeat kinase 2 (LRRK2) are the most common known cause of familial Parkinson's disease (fPD). LRRK2 is highly conserved, and *C. elegans *contains a clear ortholog, *lrk-1*. Saha et al [63] have recently shown that knockdown of *lrk-1 *sensitizes worms to the mitochondrial toxic rotenone, while pan-neuronal expression of wild type human or G^2019^S fPD LRRK2 (driven by the *snb-1 *promoter) protects against rotenone toxicity. However, transgenic LRRK2 expression also led to a preferential loss of dopaminergic neurons, with G^2019^S fPD LRRK2 being measurably more toxic than wild type LRRK2. This model should be amenable to the approaches previously used in the transgenic α-synuclein models.

Pathological forms of the prion protein (PrP) are believed to be the cause of fatal spongiform encephalies, including Creutzfeldt-Jakob disease (CJD), kuru, and Bovine Spongiform Encephaly (BSE). There is a single report describing the expression of mouse prion protein (residues 23-231) in *C. elegans *[64]. Muscle expression of a MoPrP(23-231)::CFP fusion protein (but not the CFP-only control) was found to induce visible fluorescent aggregates and a variable "Dumpy" phenotype, associated with poor locomotion and sarcomere disruption. Parallel expression of PrP expressing the P^102^L mutation associated with Gerstmann-Straussler-Scheinker disease resulted in a more severe movement deficit, while co-expression of PrP containing a dominant negative mutation (MoPrP Q167R::YFP) with MoPrP::CFP reduced apparent toxicity. However, proteinase K-resistant MoPrP could not be detected in transgenic worms, suggesting that the infectious scrapie form of PrP (PrP^sc^) was not being formed. Given the lack of PrP^sc ^formation and the fact that the transgenic constructs lack the N-terminal lipidation sequences of PrP likely important in pathology, it is unclear if this model is directly relevant to infectious prion diseases or is instead more akin to the general protein aggregation toxicity models.

## So what have we learned from *C. elegans *transgenic models?

While some of the findings from these models have been robustly demonstrated in other systems (e.g., the role of histone deacetylases in Ht::polyQ toxicity), some are clearly novel and/or have potentially broad significance to neurodegeneration. The studies of Gidalevitz et al [16] demonstrating how polyQ deposition alters the folding and function of other proteins provides the first clear experimental support for the hypothesis that aggregation-induced chaperone depletion (e.g., as suggested to explain the toxicity of ALS-mutant SOD1, [65]) can cause cellular dysfunction. The studies with worm α-synuclein models have strongly implicated vesicular function in α-synuclein toxicity [24-26]. Studies with treatments that modulate *C. elegans *lifespan have suggested why age is the primary risk factor for most neurodegenerative diseases [36,37]. The studies of Kraemer and colleagues in particular have identified a number of unexpected genes with a putative role in tau pathology [51,52,56], explicitly demonstrating the advantages of *C. elegans *models that are amenable to unbiased experimental approaches.

### Future prospects

New *C. elegans *neurodegeneration models are likely to be developed in the near future, taking advantage of both technical advantages (directed single copy transgene insertions, tetracycline-induced transgene expression), and the identification of new neurodegeneration-associated genes (e.g., TDP-43). Existing approaches have certainly not been exhausted, as genetic modifier screens have not been done to saturation, and some published large scale RNAi screens could still be expanded to full-genome screens. Clearly, some of the *C. elegans *findings still await validation in mammalian models. This is particularly important with respect to unexpected modifier genes identified in RNAi screens, given the possibility that these genes could be an artifactual result of worm-specific biology or the highly engineered nature of the model system. An important question that has not been rigorously addressed is to what degree underlying pathological processes overlap between the various neurodegeneration models. While some studies point to potential overlap of pathological processes (e.g., the common demonstration that loss of *hsf-1 *function enhances aggregation/toxicity), it remains to be determined if any suppressors in specific models also have effects in other neurodegeneration models (e.g., do *sut-1 *or *sut-2 *suppress α-synuclein or polyQ toxicity?). Technical improvements in *C. elegans *handling (e.g., the COPAS worm sorter and microfluidic worm manipulation) will also likely enable more efficient use of transgenic worm models in compound screening.

#### C. elegans neurodegeneration model check list

A sufficient number of studies have now been undertaken using transgenic *C. elegans *neurodegeneration models that a set of considerations can be established as to what is needed to establish an informative model.

#### 1) Choice of expression system

Expression of a human disease-associated protein involves the construction of a chimeric transgene employing a *C. elegans *promoter and a human cDNA. Promoters used are typically either pan-neuronal (e.g., *unc-119*, *snb-1*, *aex-3*, *rgef-1*, etc.) or targeted to specific neuronal sub-types (e.g., *mec-7*; mechanosensory neurons, *osm-10*; chemosensory neurons; *dat-1*; dopaminergic neurons, etc.). Body wall muscle-specific promoters (*unc-54 *and *myo-3*), which give strong and highly specific expression, have also been used. Promoter choice depends upon both technical considerations and specific questions being addressed. Many neurodegeneration-associated proteins are widely expressed (e.g., Abeta, tau, α-synuclein), and thus pan-neuronal promoters may be logical choices for these types of proteins. An advantage to this approach is that if pan-neuronal expression leads to toxicity in specific neuronal sub-populations (e.g., pan-neuronal expression of α-synuclein leading to specific dopaminergic neurotoxicity), it strengthens the validity of the model. However, if pan-neuronal expression of a disease-associated protein is strongly toxic, it may be impossible to recover transgenic strains in the first place. In these instances, targeting expression to non-essential neuronal sub-populations such as chemosensory neurons may be required.

Unfortunately, expression systems equivalent to the Gal4/UAS or tetracycline-induction systems developed for Drosophila have not yet been described for *C. elegans *(although this will likely change in the near future, R. Baumeister, personal communication). Thus, cell- or tissue-specific transgene expression must be individually engineered, and conditional expression has been limited to engineering heat-shock-inducible transgenes (which can be confounded by the general effects of heat shock) or the use of a temperature-sensitive mRNA surveillance system that leads to temperature-dependent levels of transgene expression [38], but does not allow on/off regulation.

#### 2) Choice of expressed proteins

***Interpretation of the effects of heterologous protein expression is completely dependent upon parallel development of the appropriate transgenic controls***. The preferable situation is to express in parallel wild type and familial disease mutant forms of a disease protein (e.g., as is available for tau, α-synuclein, SOD1, etc). Enhanced toxicity of the disease mutant forms provides support for the relevance of the transgenic model. Alternatively, it may be possible to generate disease protein variants predicted to be non-toxic (e.g., making a kinase-dead version of a disease-associated protein kinase). Demonstrating that a single amino acid change in a disease protein renders it non-toxic strongly argues against "non-specific over-expression" interpretations of transgene toxicity. As many (if not all) neurodegeneration-associated proteins are aggregation-prone, it is also possible to control for general aggregation toxicity by parallel expression of a "generic" aggregating protein such as GFP::degron [66].

(It should be noted that comparison of transgenic animals expressing "more" and "less" toxic versions of disease-associated proteins is not routinely done with mouse transgenic models, as transgenic mice are typically compared to their non-transgenic siblings. However, given the relative ease of establishing transgenic *C. elegans *strains, there is no rationale for not generating parallel control transgenic strains.)

Transgenically expressed disease proteins can conceivably be expressed with epitope (e.g., FLAG or myc) or fluorescent protein tags, although this is often unnecessary and potentially risks altering transgenic protein activities. An advantage of expressing human genes in *C. elegans *is that there is generally little cross-reactivity between specific antibodies for the human protein and worm proteins, and therefore immunoblots and/or immunohistochemistry can readily be used to follow the expression of the transgenic protein if appropriate antibodies exist. Protein fusions to GFP or other fluorescent can be very useful for rapidly establishing transgene expression and for determining the sub-cellular distribution of the transgenic protein.

#### 3) Construction of transgenic worms

There are currently three general approaches to establishing transgenic *C. elegans *strains. Transgenic strains with multicopy extrachromosomal arrays can readily be generated by gonad microinjection [67]. Irradiation and screening of these extrachromosomal transgenic strains can be used to subsequently derive completely stable, chromosomally integrated transgenic lines. Neither transgene copy number nor chromosomal insertion site can be controlled using this method. Transgenic strains can also be generated by ballistic transformation [68,69], which can directly generate single- or low copy number- transgene insertions into the chromosome. The site of chromosomal insertion is also random in this case. The recently developed Mos1-mediated single copy insertion technique (MosSCI) employs transposon excision and repair to introduce single copy transgene insertions in specific chromosomal locations [70]. This approach has great potential to generate reproducible transgenic models that will simplify comparisons of independent transgenic strains (e.g., strains expressing different disease mutant forms of a neurodegeneration-associated protein) and avoid the complications associated with high-copy transgenic arrays (high expression, germline silencing, potential genetic instability, etc).

Construction of *C. elegans *transgenic strains generally requires co-introduction of a marker transgene that enables identification of transgenic worms independent of the (usually unknown) effects of the experimental disease-associated transgene. For transgenic strains generated by the muticopy array approach, the marker transgene can be easily co-introduced by adding marker transgene plasmid DNA to the injection mix, as all injected DNA is typically assembled into the transgenic array. A plasmid encoding dominant mutant collagen [*rol-6*(*su1006*)] was commonly used as a marker transgene in early transgenic constructs [67], as introduction of this marker leads to an easily recognized "Roller" phenotype that can be seen with a standard dissecting microscope. GFP-expressing constructs (e.g. *myo-2 *promoter::GFP) are also routinely used as marker transgenes. An alternative approach is to use a plasmid that encodes a transgene that can rescue a chromosomal mutant, resulting in transgenic worms with wild type phenotypes against a background of mutant non-transgenic worms. Plasmids encoding wild type LIN-15 (rescuing a multivulval phenotype caused by recessive *lin-15 *mutations) or DPY-20 (rescuing a "Dumpy" phenotype caused by recessive *dpy-20 *mutations) have been used in this approach. Although this approach can simplify identification of transgenic worms, it does require using mutant backgrounds and has the potential confound of partial rescue of the chromosomal mutation complicating characterization of the experimental transgene phenotypes.

For ballistic- or MosSCI-mediated transformation, the marker transgene must be incorporated into the same plasmid as the experimental transgene. Expression of wild type UNC-119 protein has most commonly been used as the marker in these transformation techniques, with *unc-119 *mutant worms as the recipient. This approach is advantageous not only because the transgenic rescue of the severely uncoordinated *unc-119 *phenotype is easily seen, but because this rescue can be selected for under appropriate conditions. *unc-119 *mutant worms cannot enter the starvation-resistant dauer larval stage, and thus starvation of populations of *unc-119 *mutant worms subjected to ballistic transformation strongly enriches for transformed worms.

#### 4) Demonstration of transgene expression

The first step after establishment of a transgenic strain is demonstration of expression of the expected transgenic protein, typically done by immunoblot and/or immunohistochemistry. *C. elegans *adult worms contain ~0.4 μg of total protein, so 50-100 worms are sufficient material for one SDS-PAGE lane. Detection of transgenic proteins by immunoblot can be more difficult if expression is limited to small numbers of cells (e.g., the 8 dopaminergic neurons in *C. elegans*). Multiple protocols have been established for *C. elegans *whole mount immunohistochemistry [71], which can be used to establish transgene expression even if limited to a single cell in the worm. Transgenic proteins can of course be fused with GFP or other fluorescent proteins, which allows direct visualization of transgene expression at the risk of altering transgenic protein function.

#### 5) Establishment of transgene-associated phenotypes

Genetic (or chemical) screens require phenotypes, and thus transgenic *C. elegans *models with weak or non-existent phenotypes are less likely to be informative. In some cases, transgenic expression of disease-associated proteins can lead to strong, easily discernable phenotypes, such as uncoordinated movement in worms with transgenic neuronal expression of tau [50] or paralysis in worms with muscle expression of Aβ [31]. However, even weak transgenic phenotypes can be useful, as they can be effectively used for enhancer screens (e.g., [25]).

### Compound screening using *C. elegans *models

*C. elegans *neurodegeneration models have been used to test the effects of individual drugs (e.g., resveratrol in a polyQ model, [12]; acetaminophen in multiple PD models, [72]) or relatively small collections of compounds (e.g., subcomponents of Gingko biloba extracts in Aβ toxicity models, [39], candidate Huntington's drugs in a polyQ model, [73]). Although large numbers of replicate *C. elegans *populations can be readily grown in small volumes (e.g., microtiter wells), a previous hindrance in moving from low- to high-throughput screenings (HTS) was the requirement for manual manipulation and phenotypic scoring of worms. Recent technical advances have now overcome many of the practical limitations in using *C. elegans *models for HTS, most prominently the development of the Complex Object Parametric Analyzer and Sorter (a.k.a. the COPAS "worm sorter", [74]). This instrument can rapidly sort individual worms on the basis of length, optical density, and fluorescence, and can be used to automate both manipulation and scoring. Burns et al [75] demonstrated how this technology could be incorporated with a high-throughput digital imager and data management software for precisely scoring phenotype. This protocol was used by Kwok et al [76] to effectively screen four libraries comprised of 14,100 small molecules, which resulted in their discovery of a new L-type calcium channel antagonist. The COPAS instrument has recently been used for high-throughput neurotoxin screening [77], suggesting that this instrument should be equally applicable for screening for neuroprotective compounds in various neurodegeneration models.

For some relevant phenotypes, such as axonal regeneration after microsurgery [78] or maintenance of motorneuron synapses, subcellular observations are required. Techniques are currently being developed that may allow these challenging measurements to be automated, thereby further expanding HTS possibilities. In 2007 the Yanik group at MIT introduced a series of microfluidic devices, otherwise known as lab-on-chip technology, applicable for manipulating *C. elegans *[79]. Microfluidic devices maneuver fluids and specimens on a sub-millimeter scale via the use of layered control channels and valves. Given the size of a *C. elegans *adult (~1 mM in length and 0.1 mM in diameter), and that they are able to live out their 2-3 week lifespan suspended in liquid media, worms are an ideal animal model for this technology. These microfluidic devices can be configured with 3-dimensional imaging that allows for subcellular resolution. Additional key components of these devices are the manner with which immobilization of worms is achieved as well as the capability of continuous recirculation. A worm is captured by way of a suction channel, which restricts its movement and positions the worm in a linear fashion providing for optimal imaging. Based upon its phenotype, the animal is then either discarded or collected in a separate chamber through a series to flow valves controls. In 2008, the Hang Lu group demonstrated that integrating this lab-on-chip technology and automated multiparametric analysis could effectively identify and sort worms according to cellular and subcellular phenotypes with 95% accuracy [80]. This technology has also facilitated procedures such as those used in the *in vivo *ultrafast laser nanosurgery study conducted by Ben-Yakar and Bourgeois [81]. By integrating lab-on-chip technology and femtosecond laser nanosurgery they were able to increase the level of precision and speed with which cellular and subcellular laser ablations can be performed, suggesting the feasibility of large-scale *in vivo *neural degeneration and regeneration studies.

Despite the technical advances relative to HTS in *C. elegans*, biological considerations may still be the most important factor in the potential usefulness of *C. elegans *neurodegeneration models for drug identification. The *C. elegans *cuticle is impermeant to many compounds that readily diffuse into tissue culture cells, and thus compound entry may require ingestion along with the standard *E. coli *food source. For a given compound, there is currently no way to predict what final tissue concentration will result from a given external compound dose, so dose/responses need to be tested empirically. It can be argued that the major advantage of compound screening in *C. elegans *is also the major disadvantage: protective compounds can be identified without any assumptions about neurotoxic mechanisms, however, when protective compounds are identified, the molecular mechanism of protection may be unknown. This ignorance can be a major hurdle in further drug development, as it limits both compound optimization and predictions about possible side effects. Nevertheless, investigations of protective compounds can be a powerful approach to ultimately understanding the molecular mechanisms of neurodegeneration.

## Competing interests

The authors declare that they have no competing interests.

## Authors' contributions

DT and CDL both contributed to the writing of this manuscript and approve of the final submitted text.
